# Risk factors for the progression from acute recurrent to chronic pancreatitis among children in China

**DOI:** 10.3389/fped.2022.908347

**Published:** 2022-07-25

**Authors:** Jingqing Zeng, Jiayu Zhang, Yabin Hu, Xiumin Wang, Zhaohui Deng

**Affiliations:** ^1^Department of Pediatric Digestive, Shanghai Children’s Medical Center, Shanghai Jiao Tong University School of Medicine, Shanghai, China; ^2^Department of Clinical Epidemiology and Biostatistics, Shanghai Children’s Medical Center, Shanghai Jiao Tong University School of Medicine, Shanghai, China; ^3^Department of Pediatric Endocrinology, Shanghai Children’s Medical Center, Shanghai Jiao Tong University School of Medicine, Shanghai, China

**Keywords:** acute recurrent pancreatitis, chronic pancreatitis, pediatrics, pancreas divisum, risk factors

## Abstract

**Background:**

Risk factors for progression from acute recurrent pancreatitis (ARP) to chronic pancreatitis (CP) in children are poorly understood.

**Aim:**

To summarize the clinical characteristics of children with ARP and CP, identify the risk factors of CP, and investigate the factors associated with rapid progression from initial onset of ARP to CP.

**Methods:**

The following variables were included in the risk factor analysis: sex, age at onset, family history, pancreas or biliary tract structural abnormalities, and genetic variations. Univariate and multivariate logistic regression analyses were used to assess the risk factors of CP. The Kaplan–Meier curves of the ARP progression to CP for various risk factor groupings were constructed and compared using the log-rank test. The Cox proportional hazard regression model was fitted to estimate the hazard ratio (HR) of progression to CP for each risk variable.

**Results:**

In total, 276 children were studied, of whom 136 progressed to CP. Among them, 41 had pancreatic duct obstructive disease; 105 underwent genetic testing, of whom 68 were found to have genetic variations. Among the remaining 140 patients who did not progress to CP, 61 had biliary obstructions. Forty-three of these children underwent genetic testing, and 15 were found to have genetic variations. Risk factor analysis showed that children with gene mutations were at a higher risk of progressing to CP [odds ratio (OR) = 3.482; 95% confidence interval (CI): 1.444–8.398; *P* = 0.005]; children with pancreas divisum (PD) had a higher risk of CP than those without (OR = 8.665; 95% CI: 1.884, 9.851; *P* = 0.006). Further, children whose first ARP occurred at an older age might develop CP faster (HR = 1.070; 95% CI: 1.003, 1.141; *P* = 0.039). Children with gene mutations had a faster rate of progression to CP after onset than children without gene mutations (HR = 1.607; 95% CI: 1.024, 2.522; *P* = 0.039), *PRSS1* gene mutations were more associated (*P* = 0.025). There was no difference in the rate of progression from ARP to CP in children with PD (*P* = 0.887); however, endoscopic retrograde cholangiopancreatography (ERCP) intervention delayed the progression to CP in ARP patients with PD (*P* = 0.033).

**Conclusion:**

*PRSS1* gene mutations and PD are key risk factors for ARP progression to CP in children. PD itself does not affect the disease progression rate, but therapeutic ERCP can be beneficial to patients with ARP with symptomatic PD and delay the progression to CP.

## Introduction

Pediatric pancreatitis is often associated with high disease burden for children and families, especially for acute recurrent pancreatitis (ARP) and chronic pancreatitis (CP), with multiple emergency room visits and hospitalizations, and medical, endoscopic and surgical procedures. Moreover, patients with CP have an increased lifetime risk for pancreatic adenocarcinoma (1, 2). ARP is defined as two or more distinct episodes of acute pancreatitis (AP). Studies have shown that approximately 9–35% of children with AP develop ARP (1, 3). CP is a chronic inflammatory process that occurs in the pancreas, which leads to irreversible morphological changes and progressive impairment of exocrine and endocrine functions (4, 5). Pediatric ARP and CP are relatively rare conditions; CP has an incidence of approximately 0.5 cases per 100,000 children per year. ARP occurs frequently as a precursor of CP and is thought to be on the same disease continuum (3, 5, 6).

Most of the published findings regarding risk factors of ARP and CP are studies involving adults. However, the etiologies of ARP and CP in children are different from those in adults; they mainly involve genetic and anatomical factors in children, whereas the contribution of environmental risks, such as smoking, is negligible (1, 6, 7). A few studies have analyzed risk factors for children with CP; however, these pediatric studies are limited in that the cohorts are small, a cohort of Chinese children is rare. The factors contributing to AP recurrence and the progression from ARP to CP in children remain poorly understood and controversial.

This study aimed to characterize the demographic and clinical characteristics of children with ARP and CP to identify the risk factors of CP and the factors that accelerate the progress from the first episode of ARP to CP in Chinese children.

## Materials and methods

### Study design and participants

The clinical data of children with ARP and CP admitted to Shanghai Children’s Medical Center (SCMC) from January 2014 to March 2021 were retrospectively analyzed, including sex, age of onset, age at CP diagnosis, family history, imaging examination of the pancreatic duct or biliary tract, and genetic variation. We aimed to identify the risk factors contributing to CP and investigate the factors associated with rapid progression from the first episode of AP to CP.

Inclusion criteria: (1) children aged <18 years; (2) children was consistent with the diagnostic criteria of ARP or CP; and (3) patients who had been hospitalized at SCMC. Exclusion criteria: patients with incomplete clinical data or those without follow-up data.

The diagnostic criteria used were those of the International Study Group of Pediatric Pancreatitis: In Search for a Cure (INSPPIRE) (8). ARP is defined as two or more episodes of AP occurring at least 1 month apart with the resolution of symptoms between episodes, or complete normalization of pancreatic enzyme levels with the complete resolution of clinical symptoms between episodes of AP irrespective of the interval between episodes. Children with irreversible structural changes in the pancreas, with or without abdominal pain, exocrine pancreatic insufficiency, or diabetes, were classified as having CP.

Pancreatic and bile duct images of all patients were obtained using magnetic resonance imaging/magnetic resonance cholangiopancreatography (MRI/MRCP), computerized tomography (CT), or endoscopic retrograde cholangiopancreatography (ERCP).

Genetic testing was performed by the Genetic Diagnosis Center of SCMC. All patients were evaluated with targeted next-generation sequencing (NGS) as described previously (9). The targeted-NGS panel used in this study contains 2,742 disease-causing genes, covering all known causative genes for pancreatitis. Briefly, the sequencing library was prepared using the Agilent SureSelect XT Inherited Disease Panel (for targeted-NGS) or SureSelect XT Human All Exon V6 kit (Agilent Technologies, Santa Clara, CA, United States), and sequencing was performed by the Illumina Hiseq 2500. The sequence reads were aligned to a reference human genome (Human 37.3; SNP135) by NextGENe^®^ (SoftGenetics LLC, State College, PA, United States), and were then uploaded to the Ingenuity^®^ Variant Analysis™ (Ingenuity Systems, Redwood City, CA, United States) for filtering and annotation. The variants identified by targeted-NGS were validated by Sanger sequencing using the ABI 3700 sequencer (Applied Biosystems, Foster City, CA, United States), in indicated patient and their parents. This study was approved by the Ethics Committee of SCMC and informed consent was obtained from the patients and their parents.

### Statistical analysis

The median and interquartile range (IQR) were used to characterize non-normally distributed data, and the Mann–Whitney U test was used for between-group comparisons. Categorical variables are expressed as frequencies and percentages and were compared between groups using the Chi-square test or Fisher’s exact test. The risk factors for CP were assessed using univariate and multivariate logistic regression analyses. Using the log-rank test, we constructed and compared the Kaplan–Meier curves of disease progression from the first onset of ARP to the diagnosis of CP in different risk factor groups. The progression time, which is the time taken to progress from ARP to CP, was determined by setting the time of the first AP onset as the starting time and the time of CP diagnosis as the ending time. However, for those who did not progress to CP, the end time was the study deadline. Cox proportional hazards regression was performed by fitting the progression time to obtain the hazard ratio (HR) and 95% confidence interval (CI) of each variable affecting the progression of ARP to CP. All statistical analyses were performed using the SPSS software (version 26.0). Two-sided *P*-values < 0.05 were considered statistically significant.

## Results

### The characteristics of acute recurrent pancreatitis and chronic pancreatitis

In total, 276 children were enrolled in this study; 140 patients were diagnosed with ARP without progression to CP, and 136 were diagnosed with CP. Demographic and etiological analyses are shown in Table 1. The possibilities of male and female patients with ARP developing CP are not significantly different. The median age at ARP and CP diagnoses were 5.0 and 5.8 years, respectively. Approximately 0.7% of the children with ARP and 8.1% of those with CP had a family history of pancreatitis, and the difference was statistically significant (*P* = 0.003).

**TABLE 1:** Demographic characteristics and etiology analysis of children with ARP or CP.

Demographic characteristics	ARP	CP	*P*-value
Number	140	136	0.916
Male, *N* (%)	65 (46.4)	64 (47.1)	
Female, *N* (%)	75 (53.6)	72 (52.9)	
Age at onset			
Median (IQR)	5.0 (3.0, 8.0)	5.8 (3.5, 9.0)	0.094
≤6 years of age at first attack	91 (65.0)	77 (56.6)	0.154
Family history of pancreatitis, *N* (%)	1 (0.7)	11 (8.1)	**0.003**
Risk factors, *N* (%)			**<0.001**
Genetic*	15/43 (34.9)	68/105 (64.8)	
Biliary obstruction	61 (43.6)	10 (7.4)	
Pancreatic duct obstruction	7 (5.0)	41 (30.1)	
Medications	22 (15.7)	4 (2.9)	
Systemic disease	6 (4.3)	1 (0.7)	
Infection	3 (2.1)	0	
Trauma	1 (0.7)	1 (0.7)	
Inborn errors of metabolism	1 (0.7)	2 (1.47)	
Unknown	24 (17.1)	28 (20.6)	

Values are presented as frequencies (%) or medians (interquartile ranges). Statistically significant differences are indicated in bold.

*In total, 148 patients with completed genetic testing, including 43 children with ARP and 105 children with CP.

### Etiology analysis

Of the 276 children included in this study, 148 completed genetic testing, including 43 children with ARP and 105 children with CP. The results of the etiological analysis are presented in Table 1. Genetic and biliary obstructions were the most common causes of ARP. Fifteen of the 43 (34.9%) children with ARP who completed genetic testing had pathogenic or clinically significant probable pathogenic variants: 12 had *SPINK1* mutations, two had *PRSS1* mutations, and one had both. Of the 140 children with APR, 61 had bile duct obstruction (43.6%), including 11 with choledochal cysts, 28 with pancreaticobiliary maljunction (PBM) with bile duct stone formation, 14 with both choledochal cyst and PBM, and the remaining eight patients with tumor compression or infiltration of the bile duct, including three patients with neuroblastomas, one intraperitoneal lymphangioma, and four lymphomas.

Gene mutations and structural abnormalities in the pancreatic duct are CP’s most common etiological causes. Of the 105 children with CP who underwent genetic testing, 68 (64.8%) had genetic mutations: 40 had *SPINK1* mutation, among them, 37 cases were c.194 + 2T > C variations; 23 had *PRSS1* mutation, mainly c.365G > A (p.R122 H) and c.623 G > C (p.Gly208Ala) variations; two had both *SPINK1* and *PRSS1* mutations, one had *CFTR* mutation, one had *PKHD1* gene mutation, and one had both *PRSS1* and *CFTR* mutations. Forty-one children with CP had pancreatic duct obstructive disease, including 35 with pancreas divisum (PD) and 6 with annular pancreases.

In addition, 22 children with ARP experienced recurrent pancreatitis due to drug-related factors. One of them had a nephrotic syndrome that may be related to prednisone use, and the remaining 21 had chemotherapy drug L-asparaginase-induced pancreatitis. The results showed that other congenital metabolic diseases, systemic diseases, infections, and trauma can also cause ARP or CP, which are rare. Congenital metabolic diseases included two cases of familial hypercholesterolemia (FH) caused by an *LDLR* gene mutation and one case of hypertriglyceridemia caused by an *APOB* gene mutation; systemic diseases included three cases of Henoch–Schonlein purpura (HSP), two cases of systemic lupus erythematosus (SLE), one case of autoimmune pancreatitis (AIP) with ARP, and one case AIP with CP.

However, 24 cases (17.1%) of ARP had unknown etiology, 12 (50.0%) of whom had not undergone genetic testing; and 28 (20.6%) cases of CP had unknown etiologies, 15 (53.6%) of whom had not undergone genetic testing.

### Risk factors for chronic pancreatitis

The present study included sex, family history, gene mutations, and congenital pancreatic or bile duct structural abnormalities as in the risk factor analysis. Based on the data analysis of 136 children with CP, family history, genetic abnormalities, and structural abnormalities of the pancreatic duct or bile duct were high-risk factors for CP in the univariate analysis (*P* < 0.05). The results of the multivariate analysis showed that, compared with children without gene mutations, children with genetic abnormalities had a higher risk of developing CP (OR = 3.482, 95% CI: 1.444, 8.98; *P* = 0.005). In order to clarify the significance of a single gene mutation type for CP, we added a multivariate model 2, the *SPINK1* and *PRSS1* gene mutations were used as independent variables. There was no significant difference in the risk of developing CP in children with ARP with or without *SPINK1* mutation. However, both univariate and multivariate analysis indicated that *PRSS1* was a higher risk factor for CP (*P* = 0.026 and *P* = 0.03, respectively). Second, children with pancreatic duct obstruction, especially children with PD, had a higher risk of CP than those without PD (OR = 8.665, 95% CI: 1.884, 39.851; *P* = 0.006) (Table 2). Due to the small number of cases of annular pancreas and the large statistical bias, risk factor analysis was not performed separately.

**TABLE 2:** Logistic regression analysis results for CP risk factors in children.

Variables	Univariate model		Multivariable model 1		Multivariable model 2	
	OR (95% CI)	*P*-value	OR (95% CI)	*P*-value	OR (95% CI)	*P*-value
Sex	0.975 (0.608, 1.565)	0.916				
Age of onset	1.043 (0.977, 1.113)	0.212				
Family history	**12.232 (1.557, 96.099)**	**0.017**				
Pancreatic duct obstruction*	**17.707 (5.314, 59.007)**	**<0.001**				
Pancreas divisum	**15.825 (4.734, 52.899)**	**<0.001**	**8.665 (1.884, 39.851)**	**0.006**	**7.391 (1.660, 32.906)**	**0.009**
Biliary obstruction	**0.210 (0.111, 0.397)**	**<0.001**	0.728 (0.255, 2.079)	0.553		
Pancreaticobiliary maljunction	**0.185 (0.088, 0.388)**	**<0.001**				
Biliary cyst	**0.250 (0.104, 0.599)**	**0.002**				
Genetic variation	**3.524 (1.679, 7.395)**	**0.001**	**3.482 (1.444, 8.398)**	**0.005**		
*SPINK1*	1.478 (0.692, 3.160)	0.313				
*PRSS1*	**4.167 (1.186, 14.634)**	**0.026**			**4.121 (1.151, 14.757)**	**0.030**

*Pancreatic duct obstruction includes pancreatic division and annular pancreas. The number of annular pancreas cases was too small to be statistically analyzed separately.

Statistically significant differences (*P* < 0.05) are indicated in bolded *P*-value.

OR, odds ratio; *PRSS1*, cationic trypsinogen; *SPINK1*, serine protease inhibitor Kazal-type 1.

### Factors that contribute to the progression from the first onset acute recurrent pancreatitis to chronic pancreatitis

**FIGURE 1:**
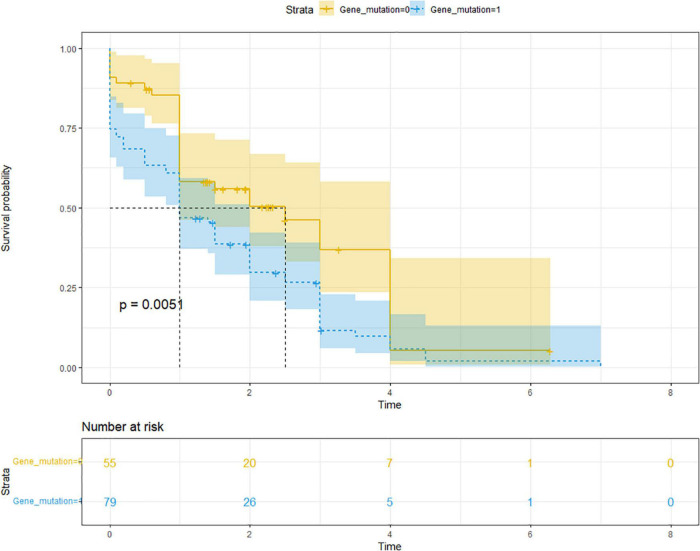
Comparison of the Kaplan–Meier curves of CP progression between those with (blue) and without (yellow) gene mutations showed a significantly faster rate of progression from ARP to CP (HR of progression was 1.607; 95% CI: 1.024, 2.522; *P* = 0.039). Median time of progression to CP was 1.0 years for those with gene mutation and 2.5 years for those without gene mutations. HR, hazard ratio; CI, confidence interval.

The mean [standard deviation (SD)] follow-up time for ARP was 2.5 (1.4) years, ranging from 0.5 to 7.0 years; the mean (SD) time for CP was 1.4 (1.6) years, ranging from 0 to 7.0 years. Of the 136 children with CP, 26 were diagnosed with CP at the first visit, and 110 (80.9%) had a history of recurrent pancreatitis. The median progression time from the initial onset of ARP to CP was 1.4 years (IQR 0.6–3.0 years). In the univariate analysis, family history (*P* = 0.015), abnormal pancreatic duct structure (*P* = 0.002), and gene mutation (*P* = 0.015) were all risk factors. Multivariate Cox hazard ratio model analysis showed that onset age and gene mutations were statistically significant. The older the age at the first onset, the faster the rate of progression to CP (HR = 1.070; 95% CI: 1.003, 1.141; *P* = 0.039), whereas gene mutation was an independent risk factor, with the occurrence of a more rapid progression to CP with genetic variation (HR = 1.607; 95% CI: 1.024, 2.522; *P* = 0.039) (Table 3 and Figure 1). We added multivariate model 2 to clarify the significance of gene mutation type for pancreatitis progression. The role of *SPINK1* in disease progression was not statistically significant; however, *PRSS1* mutation was a risk factor for the progression from ARP to CP in the multivariate analysis (*P* = 0.025) (Table 3).

**TABLE 3:** Factors that contribute to the progression of ARP to CP from first onset.

Variables	Univariate model		Multivariable model 1		Multivariable model 2	
	HR (95% CI)	*P*-value	HR (95% CI)	*P*-value	HR (95% CI)	*P*-value
Sex	0.860 (0.614, 1.205)	0.381				
Age of onset	1.038 (0.991, 1.087)	0.112	**1.070 (1.003, 1.141)**	**0.039**	**1.079 (1.015, 1.148)**	**0.015**
Family history	**2.159 (1.163, 4.007)**	**0.015**	1.536 (0.790, 2.985)	0.206	1.360 (0.868, 2.132)	0.180
Pancreatic duct obstruction	**1.803 (1.238, 2.627)**	**0.002**	0.761 (0.483, 1.198)	0.238	0.700 (0.359, 1.364)	0.295
Pancreas divisum	**1.679 (1.140, 2.471)**	**0.009**				
Annular pancreas	**2.360 (1.038, 5.364)**	**0.040**				
Genetic variation	**1.670 (1.106, 2.522)**	**0.015**	**1.607 (1.024, 2.522)**	**0.039**		
*SPINK1*	1.243 (0.835, 1.851)	0.284				
*PRSS1*	1.434 (0.912, 2.254)	0.118			**1.579 (1.060, 2.352)**	**0.025**

Statistically significant differences are shown in bold. HR, hazard ratio.

### The relationship between pancreas divisum and rapid progression to chronic pancreatitis in children

Thirty-five children with CP had PD. ERCP was performed in 15 children before progression to CP due to recurrent pancreatitis, including pancreatic duct stent placement or sphincterotomy. In comparison, ERCP treatment delayed the time to progress to CP in symptomatic PD (*P* = 0.033). Moreover, 28/35 children underwent genetic testing, and 17 cases were accompanied by gene mutations, including 10 cases of *SPINK1* variation and seven cases of *PRSS1* variation. With or without genetic variation, children with ARP accompanied by PD progressed to CP at approximately the same rate (*P* = 0.887) (Table 4).

**TABLE 4:** Relationship between pancreatic division and rapid progression of CP in children.

Variables	*N* (%)	Mean rank	*P*-value
ERCP			**0.033**
Yes	15/35 (42.9)	22.23	
No	20/35 (57.1)	14.83	
Genetic variation			0.887
Yes	17/28 (60.7)	14.68	
No	11/28 (39.3)	14.23	

Statistically significant differences (*P* < 0.05) are shown in bold. ERCP, endoscopic retrograde cholangiopancreatography.

## Discussion

This was a longitudinal retrospective study of 276 children that represented a larger population of well-characterized children with ARP or CP. In terms of age of onset, there were no sex differences between children with ARP and those with CP. The median age at first onset was approximately 5.0 years and 5.8 years for those with APR and CP, respectively. Compared with patients in other countries, patients in China are more likely to develop ARP and CP earlier (10, 11). In our study, approximately 8.1% of children with CP had a family history of pancreatitis, lower than the values reported in other countries, and the INSPPIRE reported that the prevalence of family history of pancreatitis in children with CP is approximately 23.6%, this difference may be related to ethnic differences (10).

The etiology of ARP or CP differs significantly between adults and children, with alcohol consumption and cholelithiasis being the major causes of ARP and CP in adults (12, 13). In this study, congenital bile duct or pancreatic duct structural abnormalities or gene mutations were the common causes of pancreatitis recurrence in children. However, 17.1 and 20.6% had ARP and CP, respectively, of unknown etiologies, the proportion of unknown etiology is similar to other studies on the etiology of recurrent pancreatitis (14). This relatively high proportion may be because some children with ARP or CP with genetic factors did not complete genetic testing or because of some other unknown causes of pancreatitis recurrence.

The number of drug-related risk factors in the etiological analysis of ARP in this study was higher than that in other studies (15, 16), probably because SCMC is one of the largest cancer centers in China, with many hematopathy patients receiving treatment at the center, and because asparaginase is a commonly used chemotherapy drug for acute lymphoblastic leukemia, thus increasing the proportion of cases with drug-induced ARP. Similarly, the number of patients with ARP caused by tumor compression or infiltration increased in the etiology of this study.

Most children with CP have histories of recurrent pancreatitis before diagnosis. In this study, 80.9% of the children with CP had recurrent pancreatitis before the diagnosis of CP. We focused on analyzing the high-risk factors for CP and the risk factors that promote the rapid progression of ARP to CP. This study found that genetic factors are independent risk factors for CP. The gene detection rate of CP in children was 64.8%. The *SPINK1* mutation is the most common in China, especially the C.194 + 2T > C variant, followed by the *PRSS1* mutation, but the *CFTR* mutation is less common. No *CTRC* gene mutations were detected in our study. The gene mutation results of the children in this study were consistent with those of indigenous adults but different from the results in other countries (11). In Japan, the United States, and other countries, *PRSS1* and *CFTR* mutations are more common (17–20).

We further confirmed that genetic mutations are a high-risk factor for CP. Compared with children without gene mutations, children with gene mutations associated with pancreatitis have a higher risk for CP and pancreatitis progression rate, especially those with *PRSS1* gene mutation. The findings reported by the INSPPIRE were that at least one pancreatitis-related gene mutation was found in 48 and 73% of ARP and CP patients, respectively, and *SPINK1* and *PRSS1* mutations were more closely related to CP, and children with ARP with pathogenic *PRSS1* variants rapidly progress to CP (1, 10). Overall, compared to normal children, previous studies have demonstrated the association of *SPINK1* and *PRSS1* with ARP and CP (7, 11); nonetheless, using children with ARP as a reference in this study, *PRSS1* mutations were more closely associated with CP and pancreatitis progression than *SPINK1* mutations. Another high-risk factor contributing to the recurrence of pancreatitis in children is structural abnormalities of the congenital bile duct or pancreatic duct. Previous analyses have shown that biliary diseases such as biliary obstruction can cause recurrent biliary pancreatitis. It is a common cause of recurrent pancreatitis but not a risk factor for CP in children. Structural abnormalities of the congenital pancreatic duct, especially PD, are independent risk factors for CP. However, whether PD is a high-risk factor for CP remains controversial. The results from the INSPPIRE suggest that PD may be a risk factor for ARP and CP in children, independent of genetic risk factors (21). However, it is also believed that PD alone is not a risk factor for pancreatitis but has synergistic effects with genetic factors, especially CFTR gene mutations, which can promote the development of CP (22, 23). Therefore, in this study, we conducted an independent analysis of this issue and identified PD as a risk factor for CP in children. Children with PD presenting with pancreatitis were more likely to develop CP, but this did not affect the disease’s natural history. Moreover, gene mutations and PD do not synergistically promote a faster progression to CP. However, therapeutic ERCP, such as stenting or sphincterotomy to relieve pancreatic duct obstruction, appears to provide benefit in delaying progression to CP in children with PD with recurrent pancreatitis. It would be informative to know what the results in a Chinese population are. This study also found that the age of onset was inversely correlated with the rate of disease progression, which is similar to the result reported by the INSPPIRE (10), which showed that children with ARP with an onset age >6 years progressed to CP faster than children with an onset age <6 years. Regarding the speculation about the cause of this phenomenon, it may be due to the atypical initial symptoms of pancreatic disease in some children, so there is a possibility of delayed diagnosis. Gene mutations are another risk factor for disease progression. Patients with gene mutations progress to CP more quickly after the first onset of pancreatitis.

This study focused on the risk factors for CP and for ARP’s progression to CP in a large sample of Chinese children for the first time through a longitudinal study. Some of these study’s findings are similar to those from other countries. We also obtained some interesting findings that are quite different from those of other studies. Therefore, this study expands the breadth of existing related research. However, this study had some limitations; it was a retrospective study, and all data were obtained from the SCMC, and some patients came from the pediatric cancer center of SCMC, which may cause inclusion criteria and population bias issues. However, this hospital is the only diagnosis and treatment center for children with chronic pancreatic diseases in China. Therefore, the patients came from many provinces across the country, and the present study’s findings should represent the situation across China.

In this study, we recognized that age of onset, family history, abnormal pancreatic duct anatomy, and gene mutations are essential factors to be considered in the etiological analysis of CP. We also identified risk factors associated with these factors in the progression of pancreatic disease. Overall, genetic factors and PD were independent risk factors for CP, while gene mutations were risk factors for disease progression. Therefore, for children with two or more episodes of AP or a first episode AP and family history of pancreatitis, it is recommended to perform genetic testing and imaging of the bile duct and pancreatic duct structure. This can improve our understanding of the etiology, risk factors, and the accuracy of prognostic assessments for CP.

## Conclusion

Gene mutations and congenital pancreatic anatomic variants, especially PD, are independent risk factors for CP. Children with *PRSS1* gene mutations have a faster rate of progression to CP; however, PD does not affect the disease progression rate to CP, and ERCP appears to provide a benefit in delaying progression to CP in children with PD with recurrent pancreatitis.

## Data availability statement

The original contributions presented in this study are included in the article/supplementary material, further inquiries can be directed to the corresponding authors.

## Ethics statement

The studies involving human participants were reviewed and approved by the Ethics Committee of Shanghai Children’s Medical Center. Written informed consent to participate in this study was provided by the participants or their legal guardian/next of kin.

## Author contributions

ZD and XW contributed substantially to the study’s conception and design. JYZ and JQZ contributed to the acquisition or analysis and interpretation of data. YH performed the statistical analysis. JQZ drafted the manuscript or made critical revisions related to the critical intellectual content of the manuscript. All authors have read and approved the final version to be published.
